# A retrospective analysis of adverse obstetric and perinatal outcomes in adolescent pregnancy: the case of Luapula Province, Zambia

**DOI:** 10.1186/s40748-018-0088-y

**Published:** 2018-10-17

**Authors:** Albertina Ngomah Moraes, Rosemary Ndonyo Likwa, Selestine H. Nzala

**Affiliations:** 1 Ministry of Health, Zambia National Public Health Institute, P. O. Box 30205, Lusaka, Zambia; 2 Department of Public Health, University of Zambia, P. O. Box 32379, Lusaka, Zambia

**Keywords:** Adolescent adverse obstetric perinatal outcome, Luapula, Zambia

## Abstract

**Background:**

About three in ten young women aged 15–19 have begun childbearing among the Zambian population, with adolescent pregnancy levels as high as 35% in rural areas. In 2009, Luapula reported 32.1% adolescent pregnancies. The study sought to investigate obstetric and perinatal outcomes among adolescents compared to mothers aged 20-24 years delivering at selected health facilities in Kawambwa and Mansa districts of Luapula.

**Methods:**

A retrospective analysis was carried out of all deliveries to mothers aged between 10 and 24 years for the period January 2012 to January 2013. A total of 2795 antenatal and delivery records were reviewed; 1291 adolescent mothers and 1504 mothers aged 20–24 years. Crude and adjusted odds ratios for the association between maternal age and adverse obstetric and perinatal outcomes were obtained using logistic regression models.

**Results:**

The mean age of the adolescent mothers was 17.5 years. Mothers younger than 20 years faced a higher risk for eclampsia, anaemia, haemorrhage, Cephalopelvic disproportion, prolonged labour and caesarean section. After adjustment for potential confounders, the association between maternal age and adverse obstetric and perinatal outcome diminished. Children born to mothers younger than 20 were at increased risk for low birth weight, pre-term delivery, low Apgar score and neonatal death; the risk for asphyxia, however, tended to increase with age.

**Conclusion:**

The findings demonstrate that adolescent pregnancy increases the risk of adverse obstetric and perinatal outcomes. High rates of adolescent pregnancies in Luapula province are likely as a result of the predominantly rural and poor population. Understanding the factors that contribute to the high levels of adolescent pregnancy in the region will be vital in addressing the situation and subsequently reducing the high obstetric and perinatal morbidity and mortality.

## Background

Annual global estimates of adolescent pregnancies are placed at around 16 million, about 11% of all births worldwide. An estimated 95% of these pregnancies occur in the developing world with over 50% of women in sub-Saharan Africa giving birth before the age of 20 [1]. Although accounting for only about a tenth of all births in the world, maternal conditions in adolescents produce 23% of the global burden of disability-adjusted life-years (DALYs) and 13% of all deaths from maternal conditions [2].

The majority of adolescent pregnancies are unplanned and unintended. Not only do they impact negatively on the emotional, educational and economic conditions of adolescents, but they are also associated with a high risk pregnancy [3, 4]. Pregnancy often brings a girl’s education to an end, sometimes before she finishes primary school. Many adolescents have little power to influence their own futures, let alone those of their children. Further, adolescent girls who have sex with older sexually experienced men have a higher risk of contracting HIV [1]. The role of adolescent and youth reproductive health should not be overlooked in our post-2015 bid to *ensure health and well-being for all at all ages, achieve gender equality and empower all women and girls, and ensure inclusive and equitable quality education for all* [5]*.* Combating problems related to adolescent maternal health will also impact, albeit indirectly, the bid to *end poverty in all its forms* and *end hunger and achieve food security* [6]. By improving the education, skills and prospects of pregnant adolescents, they are enabled to earn income, prevent further unwanted pregnancies and to provide for their families [7].

**Fig. 1:**
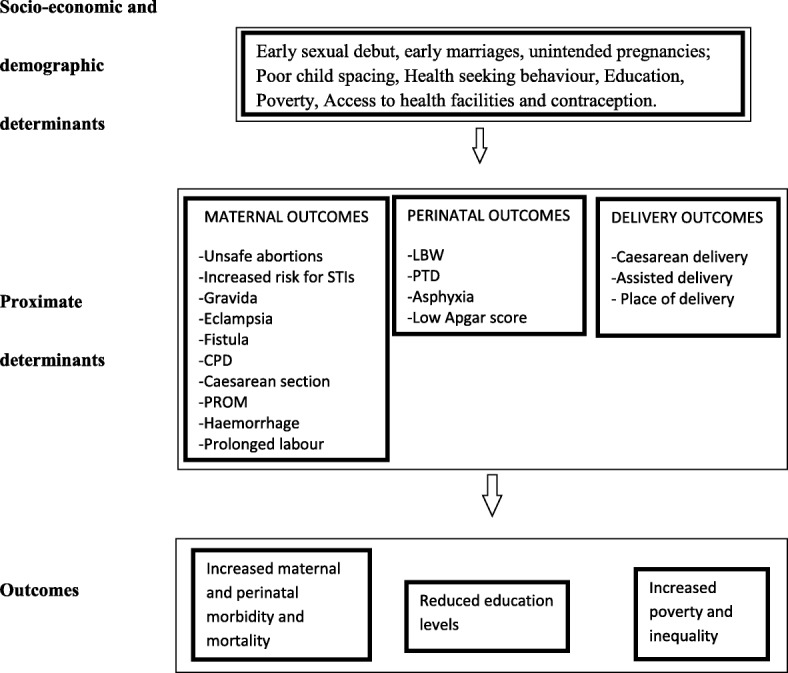
Proximate determinants framework

The framework below (Fig. 1) highlights the socio-economic, demographic and proximate determinants as well as the maternal and perinatal outcomes associated with adolescent pregnancy.

The 2002 United Nations General Assembly Special Session (UNGASS) on Children specified the need to improve care for pregnant adolescents. They declared that women, particularly adolescent expectant mothers, should have ready and affordable access to essential obstetric and perinatal care, well equipped and adequately staffed maternal health care services, skilled attendance at delivery, emergency obstetric care, effective referral and transport to higher levels of care when necessary, postpartum care and family planning in order to promote safe motherhood [8]. Often, adolescents do not receive timely antenatal care (ANC), and they have a higher risk for anaemia, malaria, HIV and other sexually transmitted infections, obstructed and prolonged labour, fistula, postpartum haemorrhage, mental disorders such as depression as well as pregnancy-related high blood pressure and its complications [9]. Obstructed labour is especially common among young, physically immature women giving birth for the first time. Those who do not die from unrelieved obstructed labour may suffer from fistula, a hole in the birth canal that leaves them incontinent and often social outcasts [10]. Up to 65% of women with obstetric fistula develop this as adolescents [11]. The prevalence of this serious morbidity is particularly high in sub-Saharan Africa. The etiology in almost all cases is neglected obstructed labour [12]. For both physiological and social reasons, mothers aged 15 to 19 are twice as likely to die in childbirth as those in their 20s, and girls under age 15 are five times as likely to die as women in their 20s [13]. Complications of childbirth and unsafe abortions are major factors leading to death in adolescents. Women aged 15–19 account for at least one fourth of the estimated 20 million unsafe abortions and nearly 70,000 abortion-related deaths each year [14].

Babies of adolescent mothers are at higher risk for asphyxia, low birth weight and premature birth thus facing an increased risk of new born health problems [15]. Given that pregnant adolescents are also more likely to smoke and drink alcohol than are older women, this can cause further problems for the child prenatally and after birth [16]. Due to the high rates of HIV/AIDS among adolescent women, children born to young mothers have an increased risk of being born with the virus [17]. Studies have shown rates of new born death to average about 50% higher to adolescent mothers when compared to mothers in their 20s [18]. Furthermore, children whose mothers die are three to 10 times more likely to die. In the first week of life, stillbirths and deaths are 50% higher among babies born to mothers younger than 20 years than among babies born to mothers 20–29 years old [19]. Deaths during the first month of life are 50–100% more frequent if the mother is an adolescent, and the younger the mother, the greater the risk [20].

**Fig. 2:**
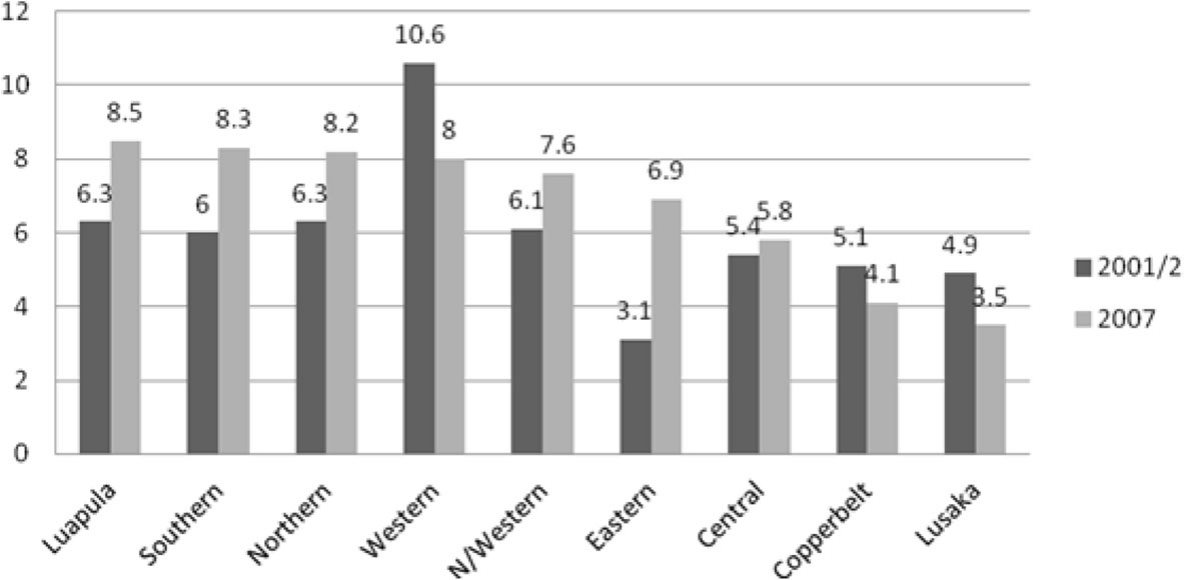
Adolescent pregnancy by province

Among the Zambian population, about three in ten young women aged 15–19 have begun child-bearing. Adolescent pregnancy levels are as high as 35% in rural areas compared with 20% in urban areas; Luapula in particular had 32.1% adolescent pregnancies [21]. Zambia’s Adolescent birth rate (ABR) is 141.2 [22], almost three times the global average of 49 per 1000 [23]. According to the 2007 DHS survey [21], at sub-regional level, Luapula province reported the highest rate of first time adolescent pregnancy (8.5%) while Lusaka had the lowest at 3.5% (Fig. 2).

It has been shown that countries with high maternal mortality ratios are generally those countries with high adolescent fertility rates [2]. Zambia’s maternal mortality ratio stands at 398 deaths per 100,000 live births [22]. The proportion of adolescent deaths contributing to this figure, however, has not been specifically documented. This is because it is difficult to attain reliable age-specific data in a well-defined population as most data are hospital-based, and the population from which they are derived remains unknown [11]. Unsafe abortions are one of the top five causes of maternal mortality in Zambia. Approximately 30% of maternal deaths are due to unsafe abortions. Induced abortions in girls younger than 18 years account for 25% of maternal deaths [21]. In a 1998 country profile by the Central Statistics Office (CSO), it was shown that around 80% of women admitted to health care facilities with complications from induced abortions were younger than 19. Many abortion-related deaths occur outside of these health institutions and usually go unreported. Ironically, Zambia has one of the most liberal abortion laws in southern Africa [24]. The infant mortality rate for children born to mothers less than 20 years old is 100 per 1000 live births, compared with 78 per 1000 live births for children whose mothers are 20-29 years old. The perinatal mortality rate for Zambia as a whole is 38 deaths per 1000 pregnancies. Perinatal mortality tends to decrease with increasing length of birth intervals and higher education level of the mother (24 deaths per 1000pregnancies) [21].

The role of maternal age and its effect on adverse obstetric and perinatal outcomes have been the subject of ongoing debate with some studies [25, 26] showing that the associations of adverse perinatal outcomes in adolescents had been confounded mainly by lack of or inadequate ANC and other socio-cultural characteristics. In contrast, other studies did not find statistically significant increase in the risk of adverse outcomes among young adolescents compared with older mothers [27, 28]. Therefore, it is unclear whether adverse obstetric and perinatal outcomes in adolescents are related to maternal age or to other factors such as lack of ANC, education level, poverty, smoking/alcohol use, as well as single parenting.

The aim of this study is to evaluate the obstetric and perinatal outcomes of adolescent pregnancies in Kawambwa and Mansa districts of Luapula Province, Zambia.

## Methods

Luapula province is located in the northern part of Zambia. It has 7 districts; the study was restricted to 2 districts- Mansa and Kawambwa. The research was based on data collected from 6 randomly selected health facilities - Mansa General Hospital, Senama Clinic, Buntungwa Clinic, Kawambwa Central Clinic, Mbereshi Mission Hospital and Kawambwa district hospital. These facilities serve as ANC clinics and delivery facilities for the population of Luapula. Data collection was conducted between December 2013 and February 2014.

The study population was mothers aged between 10 and 24 living in Mansa and Kawambwa districts of Luapula province, Zambia. Inclusion in the study group was restricted to women aged between 10 and 24 years who gave birth between January 2012 and January 2013. Adolescent mothers were categorized into 3 groups: 10-15 years, 16-17 years, and 18-19 years [29].The selection of these three age categories was considered based on the expanded age grouping of adolescence - early adolescence (ages 10–15), middle adolescence (16–17), and late adolescence (18–19) - so as to better capture the age-specific variations in the sexual, marital, and reproductive events among the age groups. Notably, sexuality and sexual activity begin around 10–15 [2] while among the Zambian population, the average age at first sexual intercourse is 17 [22]. A comparison group of 20–24 year old mothers served as the reference group for use in all comparisons in order to assess the association between maternal age and adverse maternal and perinatal outcomes. This age group was considered because it is generally regarded as safe for childbirth and is the peak child bearing age group. Only singleton deliveries were considered in order to eliminate any confounding caused by twin pregnancy.

Of the 3102 records reviewed, 2795 records had information on maternal age and were considered. Data on the number of antennal visits and obstetric and perinatal outcomes was extracted from the ANC and delivery registers at the selected health facilities.

The variables analysed were:

i. Independent variable: maternal age, defined as the age of the mother in completed years at the time of delivery.
ii. Dependent variables: adverse obstetric and perinatal outcomes for both the mother and child. The adverse maternal outcomes evaluated were eclampsia, fistulae, premature rupture of membranes (PROM), cephalopelvic disproportion (CPD), Caesarean section, anaemia, haemorrhage, sepsis, prolonged labour and maternal death. The adverse perinatal outcomes evaluated were LBW (live infant weighing< 2500 g at birth), preterm delivery (live infant delivered at < 37 weeks’ gestation), neonatal death (still birth or death occurring during the first 7 days of life), asphyxia and low Apgar scores (< 7) at 5 min.
iii. The potential confounding factors considered in the logistic regression analysis were gravida, marital status, and number of antenatal visits. Gravida was taken to be the number of pregnancies, including current pregnancy, regardless of whether the pregnancies were carried to term. Marital status was dichotomized between those who had a partner and those who did not have a partner. Antenatal visits were categorised as either poor (no visits), fair (1–2 visits) or good (3–4 visits).

The analysis was carried out using Stata version 11.0. Univariate analysis was performed using the chi-square test. Multiple logistic regression models were constructed in order to control for confounders and assess the independent association between maternal age and adverse obstetric and perinatal outcomes. The rates of adverse obstetric and perinatal outcomes were calculated for each maternal age group. Estimates of the odds ratio (OR) with 95% CI were computed as measures of association between each maternal age group and adverse obstetric and perinatal outcomes under consideration. Logistic regression models were used to control for potential confounding and to derive the adjusted ORs (aORs). Both unadjusted and adjusted odds ratios were reported. Covariates were retained in the final model based on statistical significance.

## Results

In total, 2795 delivery records were reviewed, of which 3% (*n* = 81) were 10–15 year olds, 14% (*n* = 396) were 16–17 year olds, 29% (*n* = 814) were 18–19 year olds and 54% (*n* = 1504) were 20–24 year olds (reference group). Mbereshi Mission Hospital had the highest proportion of adolescent pregnancies at 50.7%. The mean age of the adolescent mothers was 17.5 years. Table 1 below shows the distribution of adolescent births by facility.

**Table 1:** Distribution of births to adolescent mothers by facility

	Facility	Total no. Of records reviewed	No. Of births to adolescent mothers	% of adolescent mothers	Mean age of adolescent mothers	SD
1	Buntungwa Clinic	507	234	46.2%	17.6	1.53
2	Kawambwa Central Clinic	429	183	42.7%	17.4	1.02
3	Kawambwa District Hospital	501	247	49.3%	17.3	1.20
4	Mansa General Hospital	744	344	46.2%	17.5	1.08
5	Mbereshi Mission Hospital ^a^	142	72	50.7%	17.4	1.30
6	Senama Clinic	472	211	44.7%	17.8	0.91
	TOTAL	2795	1291	46.2%	17.5	1.58

^a^Mbereshi Mission Hospital was under Kawambwa District until 2014 when it was reassigned to Nchelenge District

### Maternal characteristics by age group

According to the ANC registers, the ratio of girls with a partner to those without a partner increased with age; 46.9% of those aged 10–15 reported having a partner compared to 71.8% of 15–16 year olds, 84.2% of 18–19 year olds and 94.2% of 20–24 year olds. The majority (97.1%) of adolescents aged 10–15 were gravida 1. Among those aged 16–17 years, 92.2% were gravida 1 with only 0.9% recorded as gravida 3. Similarly, among adolescents aged 18-19 years, the majority (77.8%) were gravida 1 while only 0.2% were gravida 5. Those in the reference group ranged from 32.5% in gravida 1 to 0.1% in gravida 8, with the highest proportion (45.6%) being gravida 2. Most pregnant adolescents attend at least 1 to 2 antenatal visits during their pregnancy. Less than 5% of all age groups, both adolescent and adult, under consideration failed to attend at least 1 antenatal visit. Most adolescent mothers opt to deliver from a health facility- 100% of those aged between 10 and 17, and up to 97.3% of 18–19-year olds. The maternal characteristics by age group are shown in Table 2 below.

**Table 2:** Maternal characteristics by age group

	Maternal age group			
	10–15	16–17	18–19	20–24
	N=			
Marital Status	(*n* = 32)	(*n* = 176)	(*n* = 399)	(*n* = 778)
Partner	15(46.9%)	130(71.8%)	336(84.2%)	733(94.2%)
	17(53.1%)	46(28.2%)	61(15.8%)	45(5.8%)
Gravida	(*n* = 70)	(*n* = 348)	(*n* = 667)	(*n* = 1255)
1	68(97.1%)	322(92.2%)	519(77.8%)	382(32.5%)
2	2(2.9%)	23(6.9%)	124(18.6%)	536(45.6%)
3	0(0.0%)	3(0.9%)	20(3.0%)	224(14.3%)
4	0(0.0%)	0(0.0%)	3(0.4%)	73(5.1%)
5	0(0.0%)	0(0.0%)	1(0.2%)	24(1.5%)
6	0(0.0%)	0(0.0%)	0(0.0%)	14(0.8%)
7	0(0.0%)	0(0.0%)	0(0.0%)	1(0.1%)
8	0(0.0%)	0(0.0%)	0(0.0%)	1(0.1%)
ANC	(n = 32)	(*n* = 187)	(*n* = 408)	(*n* = 776)
Poor	0(0.0%)	6(3.2%)	10(2.5%)	9(1.2%)
Fair	20(62.5%)	123(65.8%)	262(64.2%)	524(67.5%)
Good	12(37.5%)	58(31.0%)	136(33.3%)	243(31.3%)
Place of Delivery	(*n* = 22)	(*n* = 102)	(*n* = 220)	(*n* = 400)
	0(0.0%)	0(0.0%)	6(2.7%)	3(0.8%)
Hospital	22(100%)	102(100%)	214(97.3%)	397(99.2%)

A *p* value *<* 0.05 was considered statistically significant.

### The association between maternal age and adverse obstetric and perinatal outcomes

Statistical significance in the univariate analysis of the association between maternal age and adverse obstetric and perinatal outcomes was calculated using the chi-square test. Mothers under the age of 16 years were found to have the highest rates of eclampsia, haemorrhage, CPD, prolonged labour and caesarean section compared to their older counterparts. With regards perinatal outcomes, rates of LBW, pre-term delivery and low Apgar score were highest among mothers aged 10–15 years. Significant association was found between maternal age and eclampsia, fistulae, Cephalopelvic disproportion (CPD), Caesarean section, low birth weight (LBW) and perinatal mortality. However, there was no significant association between maternal age and prolonged labour, anaemia, haemorrhage, sepsis, PROM, maternal death, preterm delivery, asphyxia and low Apgar scores. The maternal and perinatal outcomes with corresponding *p* values are shown in Table 3 below.

**Table 3:** Rates of adverse obstetric and perinatal outcome by maternal age group

	Maternal age group				
	10–15	16–17	18–19	20–24	*p* value^a^
Maternal Outcomes					
	*n* = 42	*n* = 191	*n* = 358	*n* = 654	
Eclampsia (*N* = 1245)	10(23.8%)	1(0.5%)	4(1.1%)	5(0.8%)	< 0.001
Anaemia (*N* = 1245)	0(0.0%)	2(1.0%)	3(0.8%)	2(0.3%)	0.513
	*n* = 50	*n* = 245	*n* = 507	*n* = 915	
Haemorrhage (*N* = 1717)	3(6.0%)	2(0.8%)	9(1.8%)	16(1.8%)	0.089
	*n* = 49	*n* = 209	*n* = 405	*n* = 724	
Sepsis (*N* = 1387)	0(0.0%)	2(1.0%)	4(1.0%)	4(0.6%)	0.751
	*n* = 22	*n* = 102	*n* = 220	*n* = 400	
Fistulae (*N* = 744)	0(0.0%)	0(0.0%)	4(1.8%)	19(4.8%)	0.032
PROM^b^ (*N* = 744)	0(0.0%)	1(1.0%)	4(1.8%)	4(1.0%)	0.767
	*n* = 30	*n* = 156	*n* = 369	*n* = 661	
CPD^c^ (*N* = 1216)	11(36.7%)	29(18.6%)	39(10.6%)	64(9.7%)	< 0.001
	*n* = 40	*n* = 217	*n* = 446	*n* = 542	
Prolonged labour (*N* = 1245)	3(7.1%)	8(4.2%)	19(5.3%)	25(3.8%)	0.576
	*n* = 57	*n* = 263	*n* = 554	*n* = 985	
Caesarean section (*N* = 1859)	25(43.9%)	70(26.6%)	125(22.6%)	187(19.0%)	< 0.001
	*n* = 22	*n* = 102	*n* = 220	*n* = 400	
Maternal death (*N* = 744)	0(0.0%)	2(2.0%)	2(0.9%)	8(2.0%)	0.682
Perinatal outcomes					
	*n* = 70	*n* = 332	*n* = 706	*n* = 1258	
Low birth weight (*N* = 2366)	12(17.1%)	52(15.7%)	103(14.6%)	140(11.1%)	0.035
	*n* = 37	*n* = 174	*n* = 416	*n* = 731	
Pre-term delivery (*N* = 1358)	2(5.4%)	9(5.2%)	15(3.6%)	18(2.5%)	0.239
	*n* = 30	*n* = 156	n = 369	n = 661	
Asphyxia (*N* = 1216)	0(0.0%)	2(1.3%)	7(1.9%)	4(0.6%)	0.248
	*n* = 57	*n* = 263	n = 554	n = 985	
Low Apgar score (*N *= 1859)	9(15.8%)	38(14.4%)	61(11.0%)	92(9.3%)	0.062
Neonatal death (*N* = 1859)	4(7.0%)	7(2.7%)	38(6.9%)	39(4.0%)	0.018

^a^chi-square test was used to identify between-group differences. Fischer’s exact test was used when the number of outcomes was small

^b^PROM = Premature Rapture of membranes

^c^CPD = Cephalopelvic disproportion

### Multiple Logistic Regression models of risk factors for adverse obstetric and perinatal outcomes in adolescent mothers

List-wise deletion method was used for missing data in the logistic regression model; only records with available data on each variable were analysed. Compared to mothers aged 20–24, young adolescent mothers were found to face higher odds for eclampsia, anaemia, haemorrhage, sepsis, cephalo-pelvic disproportion, prolonged labour and caesarean section, although not all were statistically significant. The odds of anaemia and sepsis were higher in older adolescents (16–19) but not statistically significant. Young maternal age was associated with higher odds for low birth weight, asphyxia, pre-term delivery, low Apgar score and neonatal death; the odds for asphyxia, however, tended to increase with maternal age.

Logistic regression models were used to control for the selected confounders and to derive the adjusted ORs (aORs), however, this was only possible for certain variables due to the small number of outcomes. These are shown in Tables 4 and 5 below for maternal and perinatal outcomes respectively. When adjusted for confounding, the association between maternal age and adverse obstetric and perinatal outcome diminished. Mothers aged 10–15 still had higher odds of eclampsia, CPD, and prolonged labour Children born to mothers younger than 18 had higher odds of having LBW.

**Table 4:** Logistic regression models of risk factors for adverse maternal outcomes^a^

Maternal outcomes	Maternal age group	Crude Odds ratio (95% CI)	Adjusted Odds ratio (95% CI)
Eclampsia	10–15	*40.563 (13.094–125.651)*	*30.503 (7.802–119.247)*
(N = 1245)	16–17	0.683 (0.079–5.883)	0.459 (0.049–4.312)
	18–19	1.467 (0.391–5.497)	0.179 (0.019–1.697)
Anaemia	10–15	Non-estimable	Non-estimable
(N = 1245)	16–17	3.450 (0.483–24.652)	7.645(0.439–133.114)
	18–19	2.755 (0.458–16.563)	1.252 (0.093–16.765)
Haemorrhage	10–15	*3.586 (1.010–12.739)*	0.071 (0)
(N = 1717)	16–17	0.463 (0.106–2.025)	Non-estimable
	18–19	1.015 (0.446–2.315)	0.022 (0.001–0.437)
Sepsis (N = 1387)	10–15	Non-estimable	0.466 (0)
	16–17	1.739 (0.316–9.561)	0.475(0)
	18–19	1.796 (0.447–7.218)	0.250(0.013–4.729)
Fistulae (N = 744)	10–15	Non-estimable	Non-estimable
	16–17	Non-estimable	Non-estimable
	18–19	0.371 (0.125–1.106)	Non-estimable
PROM (N = 744)	10–15	Non-estimable	Non-estimable
	16–17	0.980 (0.108–8.865)	1.804 (0.175–18.588)
	18–19	1.833 (0.454–7.404)	0.712 (0.108–4.685)
CPD (N = 1216)	10–15	*5.401 (2.461–11.852)*	1.604 (0.721–3.569)
	16–17	*2.130 (1.320–3.438)*	0.687 (0.416–1.136)
	18–19	1.102 (0.724–1.678)	0.471 (0.301–0.736)
Prolonged labour	10–15	1.488 (0.432–5.126)	2.569 (0.710–9.289)
(N = 1245)	16–17	0.345 (0.119–0.995)	0.397 (0.136–1.155)
	18–19	0.862 (0.479–1.552)	0.928 (0.512–1.682)
Caesarean section	10–15	*3.334 (1.930–5.760)*	0.082 (0.011–0.616)
(N = 1859)	16–17	*1.548 (1.128–2.124)*	0.124 (0.044–0.355)
	18–19	1.243 (0.964–1.605)	0.048 (0.015–0.151)
Maternal death	10–15	Non-estimable	Non-estimable
(N = 744)	16–17	0.980 (0.205–4.687)	1.242 (0.251–6.142)
	18–19	0.449 (0.095–2.136)	0.207 (0.031–1.386)

^a^The covariates included in the logistic regression model were marital status, gravida and ANC

**Table 5:** Logistic regression models of risk factors for perinatal outcomes^a^

Perinatal outcomes	Maternal age group	Crude Odds ratio (95% CI)	Adjusted Odds ratio (95% CI)
Low birth weight (N = 2366)	10–15	1.652 (0.866–3.152)	1.418 (0.417–4.826)
	16–17	*1.483 (1.051–2.092)*	1.455 (0.811–2.611)
	18–19	*1.364 (1.038–1.792)*	0.771 (0.461–1.287)
Pre-term delivery (N = 1358)	10–15	2.263 (0.505–10.142)	0.463 (0.038–5.627)
	16–17	2.161 (0.954–4.895)	0.376 (0.078–1.815)
	18–19	1.482 (0.739–2.972)	0.435 (0.119–1.596)
Asphyxia (N = 1216)	10–15	Non-estimable	Non-estimable
	16–17	2.133 (0.387–11.755)	0.717 (0.051–10.048)
	18–19	3.176 (0.924–10.926)	0.361 (0.028–4.706)
Low Apgar score (N = 1859)	10–15	1.820 (0.865–3.828)	0.034 (0.002–0.401)
	16–17	*1.639 (1.093–2.458)*	0.263 (0.097–0.710)
	18–19	1.201 (0.853–1.690)	0.087 (0.030–0.249)
Neonatal death (N = 1358)	10–15	1.831 (0.631–5.313)	Non-estimable
	16–17	0.663 (0.293–1.500)	Non-estimable
	18–19	*1.786 (1.128–2.828)*	0.025 (0.002–0.265)

OR = 0 no outcome event for the age group

OR = 1 exposure does not affect odds of outcome

OR > 1 exposure associated with higher odds of outcome

OR < 1 exposure associated with lower odds of outcome

^a^The covariates included in the logistic regression model were marital status, gravida, and ANC

## Discussion

The adolescent birth rate was found to be 461.9 per 1000 deliveries to mothers aged between 10 and 19 years, which is three times higher than the national ABR of 141.2 [22]. This is not surprising given that literature has shown that among rural and poor populations the rates of adolescent pregnancy do tend to be higher. The majority (69.2%) of all the adolescent mothers were in their first pregnancy. The youngest mother in the study population was 11 years old at the time of delivery. The findings demonstrate a clear trend of higher risk of adverse outcomes, particularly in mothers below 16 years. Given that adolescent mothers are more likely than older mothers to have sociodemographic characteristics associated with adverse outcomes of pregnancy, the confounding effects of marital status, gravida, adequacy of ANC and place of delivery were accounted for.

The population under consideration for this study allowed for a comparison of adolescent and adult obstetric and perinatal outcomes, something few studies have done. Controlling for confounding factors and the relatively homogeneous population of women studied further strengthen the findings of this study. Unlike most studies on adolescent pregnancy which focus on adolescents aged 15 to 19 years, − with information about pregnancy at younger ages usually only appearing in aggregate statistics -, this study considered very young maternal age (10-15 years). Data was obtained from the ANC and delivery registers, which consisted of information recorded during ANC visits and immediately following delivery respectively. Some records that did not have available data on each variable were dropped using list-wise deletion (complete case analysis). This meant not all the information collected was used and effectively lowered the sample size and thus the statistical power was reduced and some bias introduced in the estimates. Further bias in the reported estimations was introduced from the use of the maximum likelihood estimation of the logistic model. The study did not take into consideration socio economic indicators such as education and employment status as well as behavioural risk factors such as maternal smoking and alcohol consumption.

Eclampsia in adolescents is of critical importance considering the untimely access and usage of ANC services which can be used to monitor and manage the condition [11]. If left unchecked, eclampsia poses significant risks to both the mother and baby and can lead to death. According to the findings of this study, adolescent mothers were forty times more likely to develop eclampsia compared to the reference group of mothers aged 20–24 years. This is in line with the findings of other studies [30–32]. It was noteworthy to find that most adolescents do not attend the prescribed 3–4 ANC visits during their pregnancy; at most, they attend an average of 1–2 visits. Given that adolescent pregnancy has been shown to be high risk, this is far from ideal.

Furthermore, young maternal age was found to be a risk factor for haemorrhage. This may be attributed to an increased risk of placenta abruption in younger mothers [33]. Haemorrhage has been shown to be dangerous, particularly in adolescent mothers presenting with anaemia [34]. This study also found that younger mothers are five times more at risk for CPD than older mothers. In a similar study [35], adolescent mothers had an almost nine times higher risk for CPD than those above 20 years. CPD has been linked to the immaturity of the pelvic bones and birth canal in younger mothers. This immaturity has also been linked to increased risk of prolonged and obstructed labour, episiotomy, and use of forceps [31].

The study also showed an increased risk for prolonged labour and caesarean section before adjustment for confounding. This data is in line with findings of other studies [36, 37]. Caesarean sections today are a timely operative procedure that often save the lives of mothers and babies. Available literature on caesarean sections in adolescents is conflicting with some studies [38, 39] reporting higher rates among adolescents and other reporting lower or similar rates in adolescents compared to older mothers [40, 41] This makes it difficult to draw a clear conclusion, especially given that indications for a caesarean section are quite subjective among obstetricians. Caesarean sections have been linked to intra- and post-operative complications such as placenta previa and placenta accreta, hysterectomy, and bladder and bowel injury [42].

Very little information was available on the levels of fistulae. The complete number of fistulae cases were hard to detect as the only centre in the province where women can access treatment on a regular basis is Mansa General Hospital. Women from all over the province with this condition have to wait for indefinite periods to get access to the service. Some patients are lost to follow up due to being referred from one hospital to another, and in cases where funds are unavailable, this is at their own expense. For the period January 2012 to December 2012, 23 women underwent corrective surgery for Fistulae. Of these, 19 were between 20 and 24, and 4 were aged 18–19 years. The available data, however, does not indicate when the condition developed and is in no way representative of the complete picture.

The maternal mortality rate among adolescent mothers was 3 deaths per 1, 000 live births. In comparison to the findings of a previous study in Mansa [43], this shows a marked decrease in reported mortality, possibly as a result of policies and intervention introduced in the early 2000s to address the high levels of maternal mortality in the country. There was no marked difference in the mortality among adolescent mothers and those aged 20-24 years; both age groups recorded a 2% mortality rate. Literature has shown that the increased risk of maternal death in adolescent mothers has been linked to complications during and following pregnancy and childbirth. These include eclampsia, haemorrhage, sepsis and unsafe abortions. Other complications may exist before pregnancy but are exacerbated during pregnancy [44].

Literature has shown that adolescent mothers continue to grow during pregnancy and are therefore in competition with the developing foetus for nutrients, to the detriment of the foetus [45]. According to the findings of this study, compared with infants born to mothers aged 20 to 24 years, those born to women aged 15 years or younger had about 50% risk of low birth weight. They were also faced with a higher risk of pre-term delivery, low Apgar score and neonatal death. These findings are in line with those of other studies [46–48]. It has been suggested that the higher incidence of low birth weight among adolescent mothers is likely linked to pre-term delivery [49]. Notwithstanding, the risk diminished when it was adjusted for confounders, suggesting that the increased risk of early pre-term delivery, low Apgar score and neonatal death among the youngest adolescents may be explained by the access to ANC, partner status and gravida. Only low birth weight remained a risk factor for young maternal age even with the effect of confounding. This has been shown to be as a result of the competition for nutrients between the growing mother and the foetus and may also be attributed to inadequate weight gain during the pregnancy [50, 51]. However, this was not taken into consideration during this study.

The neonatal mortality rate among adolescents was 38 deaths per 1, 000 live births. Neonatal death was highest among very young mothers (10-15 years old) with 7% mortality recorded, almost double that found among mothers aged 20-24 years. Previous investigations of perinatal mortality in adolescent pregnancy have produced conflicting results. Some studies [31, 36] have found an increased risk of neonatal mortality among adolescent mothers, whereas others found no increase [52, 53].

The high prevalence of adolescent births in the region is likely as a result of the rural setting where high poverty levels and lower educational levels are the order of the day, with 64.91% of the population living in abject poverty, 83.8% literacy rates among 15–24 year olds and a ratio of 0.74 girls to boys in secondary education [54]. The number of adolescents reporting having a partner during ANC was alarming, with as many as 46.9% of 10–15 year olds registered as married in the ANC registers. According to a qualitative study by UNICEF [55], early and forced marriages are rife in Luapula Province, with an estimated incidence of 70% early pregnancy and under-age marriage among adolescents.

The situation is so dire that the Zambian Government has put in place policies that allow adolescents access to a full range of sexual and reproductive health services, including condoms and other means of contraception. The National Population Policy which seeks the reduction of the high levels of fertility, particularly adolescent fertility [56]. Other policies include the National Youth Policy and the National Reproductive Health Policy. However, the concept of adolescent friendly health services (ADFHS) introduced by WHO to define appropriate and convenient health services for adolescents has not been fully implemented in Zambia. Some facilities have youth friendly corners established which serve as entry points of access to care. Although these offer similar services to ADFHS, they target youths as a whole, which comprise different age groups with different health needs and as such adolescents’ needs are not specifically targeted [57]. The Saving Mothers Giving Life (SMGL) program, piloted in Zambia in 2012, has intensified efforts to strengthen health services focused on the critical period of labour, delivery and the first 48 h post-partum. Since its launch, the efforts of SMGL have helped improve maternal health outcomes in Zambia. The United Nations Population Fund (UNFPA) launched its manual for healthcare providers from low-and middle-income countries involved in the prevention and management of fistula in August of 2011 and has made great strides, prior to which Zambia did not have a standard training manual. Despite the numerous efforts of government, UNFPA and other stakeholders as well as programs such as SMGL, there is still a clear and pressing need for continued advocacy against teenage marriage as well as at adolescent reproductive health services and school curriculums. One of the major challenges facing the provision of adolescent health services in Zambia is financing. Currently, even though these activities are included in the action plans and budgets, youth friendly services are yet to receive budgetary allocations, and therefore when funds are disbursed, adolescent health activities are not prioritised.

## Conclusion

Adolescent pregnancy plays a crucial role in maternal and perinatal health; improvements in reproductive health cannot be complete without improvements in adolescent health. According to current statistics, Zambia’s maternal and perinatal mortality rates are amongst the highest in the region. The findings of this study highlight the high levels of adolescent maternal and perinatal mortality and morbidity in Luapula province. This will assist in the promotion of programs and policies advocating for the improvement of adolescent maternal-perinatal health as well as build on the currently available literature.

Adolescent pregnancy, especially in mothers younger than 16 years, increases the risk of adverse obstetric and perinatal outcomes. Efforts to curb the high number of adolescent pregnancies through policy initiatives as well as reproductive health education and better antenatal and obstetric care targeted at adolescents have been shown to play a major role in reducing overall maternal and perinatal morbidity and mortality rates. Despite these efforts and interventions, adolescent pregnancy and its associated adverse outcomes continue to be a problem in the province. Given this trend, it would be imperative to tailor interventions to reduce unintended pregnancies and address adolescent health needs to the specific population. Recognising the existence of avoidable factors that play a key role in the outcomes of adolescent pregnancies, such as access to ANC, must be the first step in designing and implementing intervention programmes. Where possible, evaluations of these interventions that follow the adolescents into adulthood should be performed.

## Abbreviations

ADFHS: Adolescent Friendly Health Services
AIDS: Acquired Immuno-Deficiency Syndrome
ANC: Antenatal care
CPD: Cephalopelvic disproportion
CSO: Central Statistics Office
DALY: Disability-Adjusted Life-Years
HIV: Human Immuno-deficiency Virus
LBW: Low birth weight
MDGs: Millennium Development Goals
MMR: Maternal mortality ratio
MoH: Ministry of Health
PROM: Premature rapture of membranes
SMGL: Saving Mothers Giving Life
UNDESA: United Nations Department of Economic and Social Affairs
UNFPA: United Nations Population Fund
UNZABREC: University of Zambia Biomedical Research Ethics Committee
WHO: World Health Organisation
YFC: Youth Friendly Corner
ZDHS: Zambia Demographic Health Survey
